# Supplementary figures and images for: Varicella zoster virus encodes a viral decoy RHIM to inhibit cell death
Source: PLoS Pathog. 2020 Jul 10;16(7):e1008473. doi: 10.1371/journal.ppat.1008473 (PMC7375649; doi:10.1371/journal.ppat.1008473)

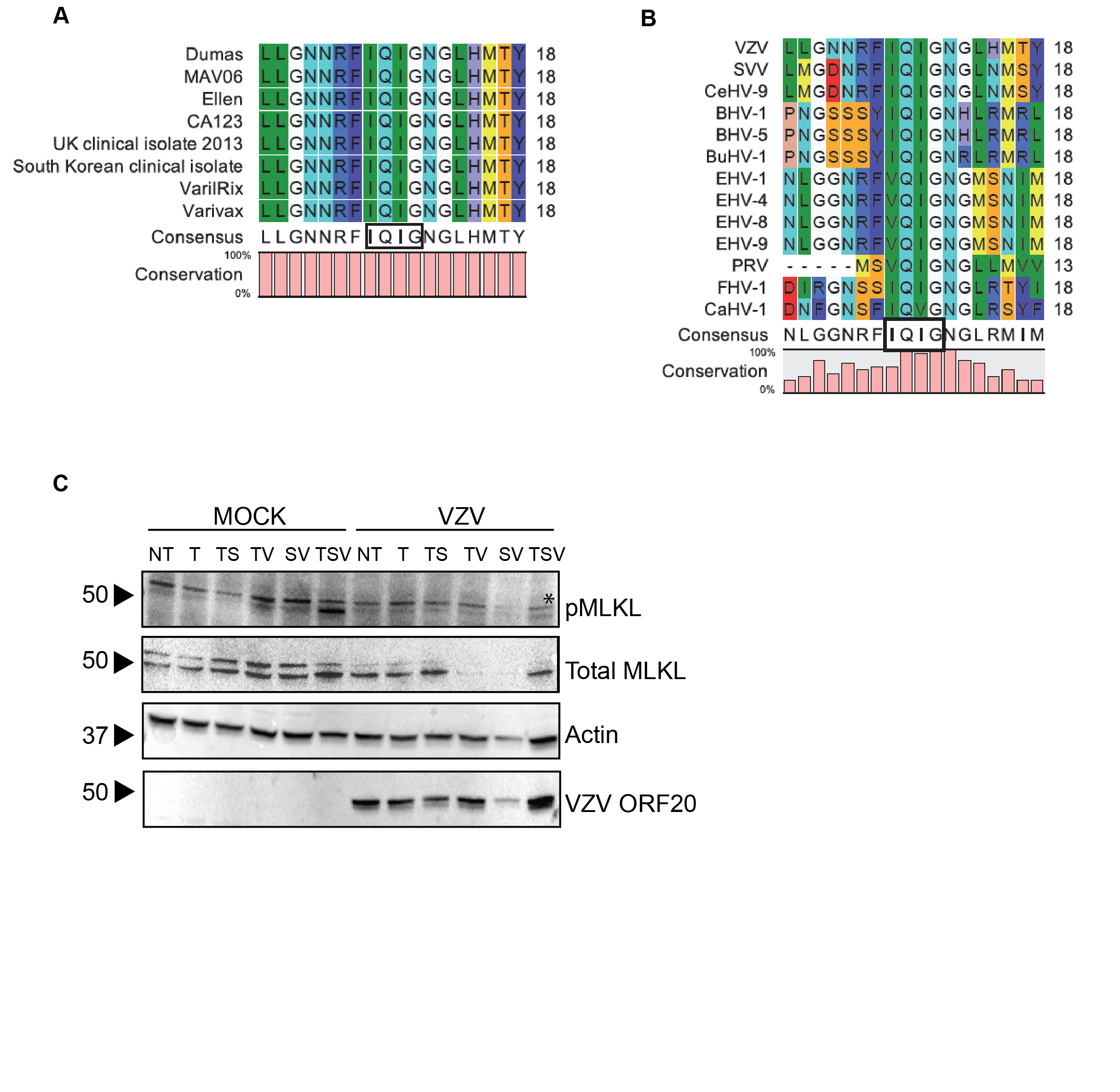

Supplement: S1 Fig — (A) Amino acid sequence alignment of various VZV strains showing the ORF20 RHIM region and indicating the percentage of conservation and consensus sequence. Accession numbers of VZV sequences used: X04370.1, JF306641.2, JQ972913.1, DQ457052.1, KX262866.1, KU926322.1, DQ008355.1, DQ008354.1 The RHIM core is boxed (B) Amino acid sequence alignment of the RHIM identified in VZV ORF20 (Dumas) with potential RHIMs in the capsid triplex subunit 1 from other Varicelloviruses, Simian varicella virus (SVV), Cercopithecine herpesvirus 9 (CeHV-9), Bovine herpesvirus (BHV) -1 and -5, Bubaline herpesvirus 1 (BuHV-1), Equine herpesvirus (EHV) -, -4, -8 and -9, Pseudorabiesvirus (PRV), Feline herpesvirus -1 (FHV1), and Canine herpesvirus -1 (CaHV-1). (C) Immunoblot analysis of mock and VZV-infected HT-29s following with TNF (T; 30 ng/ml), BV-6 (S; 1 μM), z-VAD-fmk (V; 25 μM). * represents a non-specific band, arrows indicate protein size markers. (TIF) [file ppat.1008473.s001.tif]

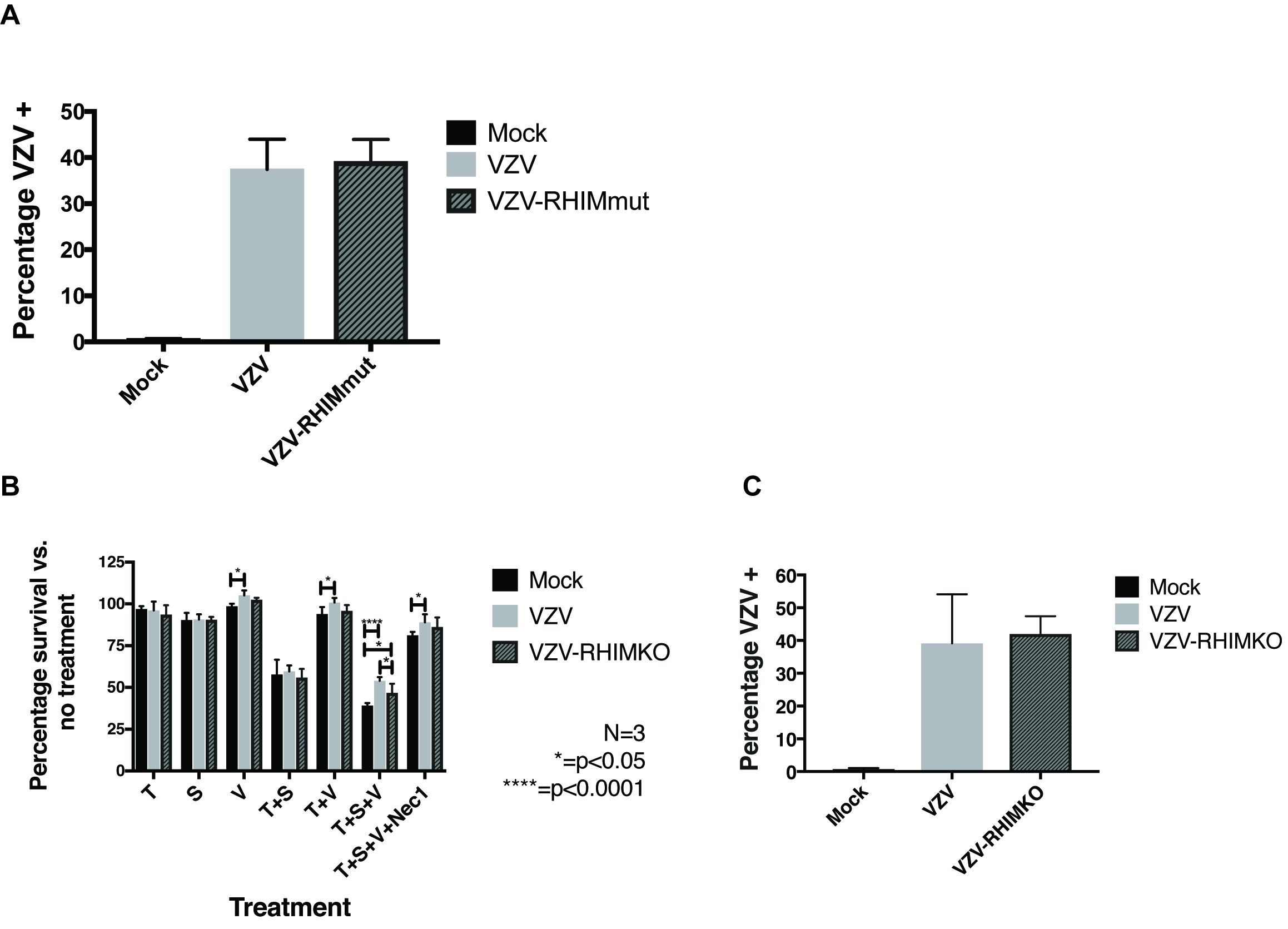

Supplement: S2 Fig — (A) Average percentage of VZV-infected cells in the parental and VZV RHIM mutant virus HT-29 cultures at the beginning of each viability assay as determined by flow cytometry staining for the gE:gI glycoprotein complex. Error bars show standard error of the mean, from 4 independent replicates. (B) Viability of mock, VZV and VZV RHIM deleted (VZV-RHIMKO) virus infected HT-29s (72 h post-infection) following treatments with TNF (T; 30 ng/ml), BV-6 (S; 1 μM), z-VAD-fmk (V; 25 μM) and necrostatin-1 (Nec1; 30 μM) alone or in combination as indicated. Data was normalised to DMSO only control. Error bars show standard error of the mean, from 3 independent replicates and statistical significance was determined using a two-way ANOVA. C. Average percentage of VZV-infected cells in the parental and VZV RHIM deleted virus HT-29 cultures at the beginning of each viability assay as determined by flow cytometry staining for the gE:gI glycoprotein complex. Error bars show standard error of the mean, from 3 independent replicates. (TIF) [file ppat.1008473.s002.tif]

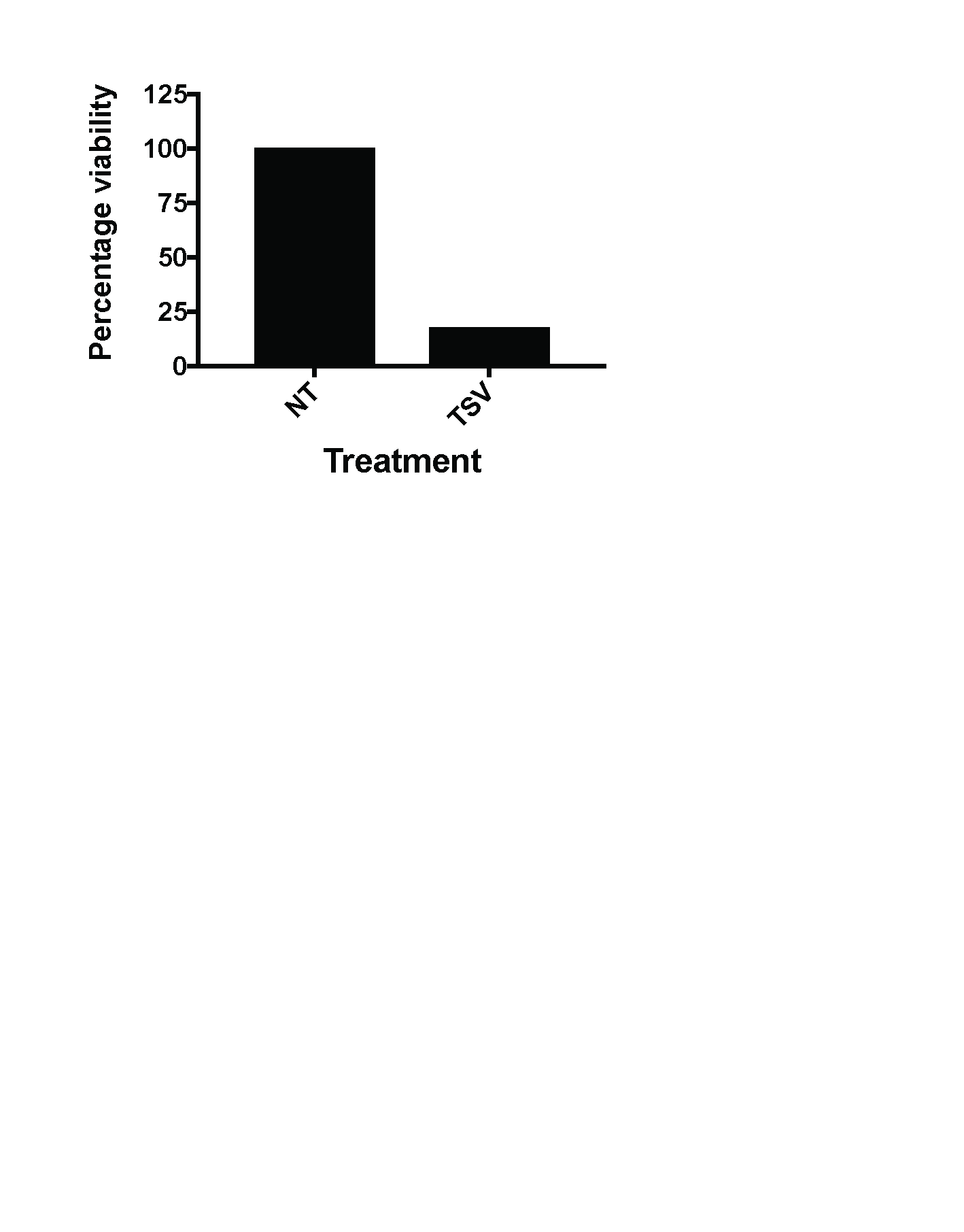

Supplement: S3 Fig — ZBP1 expressing HT-29s were treated with DMSO (No treatment, NT) or with TNF (T; 30 ng/ml), BV-6 (S; 1 μM), z-VAD-fmk (V; 25 μM) for 18 h then cell viability measured using the Promega Cell Titre Glo2 assay. (TIF) [file ppat.1008473.s003.tif]

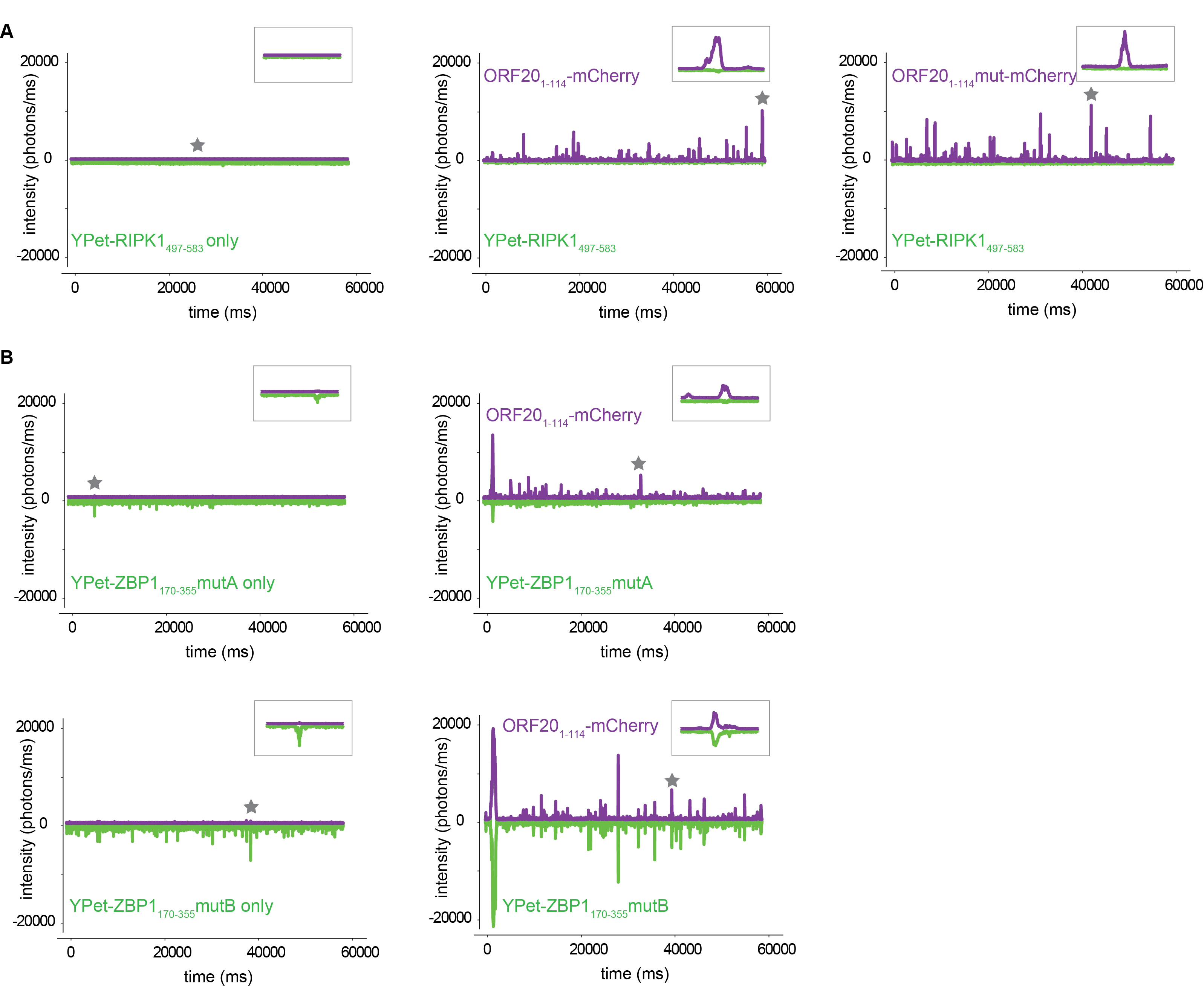

Supplement: S4 Fig — Representative confocal fluorescence spectroscopy time profiles collected from (A) ORF201-114-mCherry and ORF201-114mut-mCherry with YPet-RIPK1497-583, and (B) YPet-ZBP1170-355mutA or YPet-ZBP1170-355mutB fusion proteins with ORF201-114-mCherry. Proteins were incubated alone or mixed in pairs under conditions that allow co-assembly. The proteins present in each mixture are indicated for each part of the figure. Inserts show detail of 1 s of the dual fluorescence recordings, at the time indicated by a star on the full time trace. (TIF) [file ppat.1008473.s004.tif]

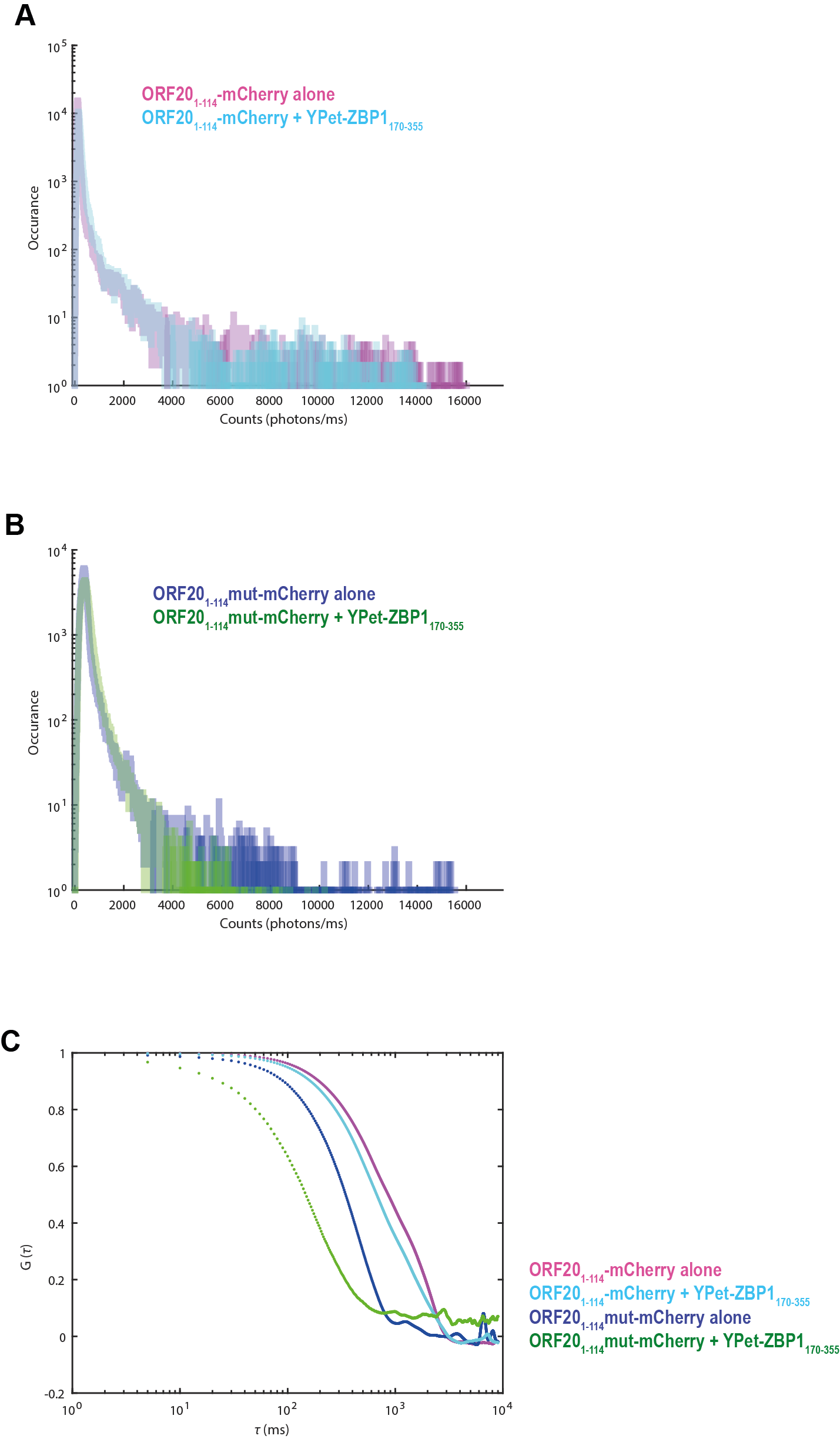

Supplement: S5 Fig — (A) Photon count histogram of fluorescence intensity detected in mCherry emission channel reflects oligomer particle size distribution over a 3 min period for ORF201-114-mCherry alone or in combination with YPet-ZBP1170-355. (B) Photon count histogram of fluorescence intensity detected in mCherry emission channel reflects oligomer particle size distribution over a 3 min period for ORF201-114mut-mCherry alone or in combination with YPet-ZBP1170-355. (C) Fluorescence correlation analysis reveals distribution of particles of different sizes, reflected by correlation coefficient (tau). (TIF) [file ppat.1008473.s005.tif]

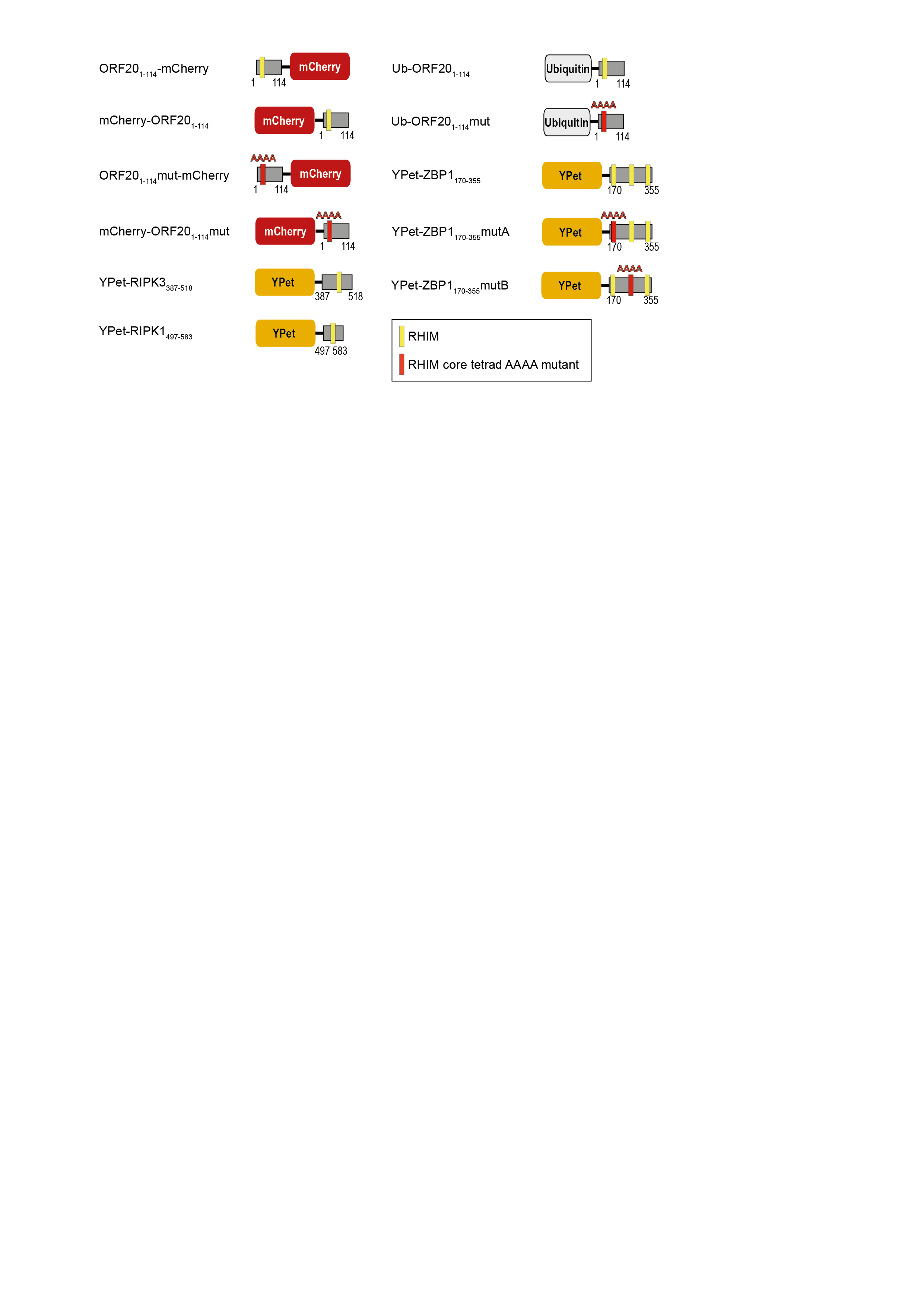

Supplement: S6 Fig — (TIF) [file ppat.1008473.s006.tif]
