# Supplementary material for: Varicella zoster virus encodes a viral decoy RHIM to inhibit cell death
Source: PLoS Pathog. 2020 Jul 10;16(7):e1008473. doi: 10.1371/journal.ppat.1008473 (PMC7375649; doi:10.1371/journal.ppat.1008473)
Supplement: Attachment — Submitted filename: Steain et al., reply to reviwer.pdf [file ppat.1008473.s007.pdf]

THE UNIVERSITY OF
SYDNEY

May 22, 2020

Dear Professor Mocarski,

Thank you for the opportunity to revise our manuscript “Varicella zoster virus encodes a decoy RHIM to inhibit cell death”. We would like to thank the reviewers for their insightful comments.

Unfortunately, due to the current coronavirus global pandemic we have not been able to access our laboratory to perform any new experiments as requested, and the current situation is unlikely to change significantly in the foreseeable future. However, we have been able to address many of the queries and comments in light of results we had previously not included. We believe we have made changes that significantly improve the manuscript. This includes including extra data requested by both Reviewer#1 and Reviewer#2 with respect to replication of the different viruses used in the study. We have also added data to address Reviewer#2, point 2, and provide data to address Reviewer#3, point 1. Please see our detailed responses and explanation of changes made to the manuscript below. We have also submitted updated versions of the manuscript +/- highlighted changes as requested.

Our work adds to the rapidly growing body of evidence around the importance of ZBP1 as a sensor of foreign nucleic acid. We hope that you will find that we have addressed all of the major concerns raised by the reviewers in this revised and improved manuscript and will now find it suitable for publication in PLoS Pathogens.
Yours sincerely,

Megan Steain and Allison Abendroth

Infectious Diseases and Immunology
School of Medical Sciences

Faculty of Medicine and Health
Lvl 5E, Charles Perkins Centre D17
The University of Sydney
NSW 2006 Australia

T +61 2 9351 6178
F +61 2 86271608
E megan.steain@sydney.edu.au
sydney.edu.au

ABN 15 211 513 464
CRICOS 00026A

Major issues raised:

Reviewer #1:
1. *In this article, the authors described the importance of ORF20 for protection from programmed cell death and these results clearly show the interaction between the ORF20 RHIM domain and ZBP1. However, the data in infected cells is lacking. The authors should show the importance of the RHIM domain in ORF20 for viral growth in normal cells such as MRC-5 cell.*

Whilst we have not been able to perform a time course experiment looking at the mutant virus infection in cells typically studied with VZV due to our inability to perform experiments at the moment due to Covid-19 related restrictions, there are several factors that provide supporting evidence that the mutations do not impact viral replication in cells lacking ZBP1. When the viruses were originally recovered from BACs in ARPE-19 cells, the two mutant viruses (VZV-RHIMmut and VZV-RHIMKO) all produced plaques at the same size and rate as the parental strain (all 3 were recovered from BACs in one experiment). Additionally, when passaging the viruses in APRE-19 cells, the same split rate was used over several passages as the parent pOKA strain and following passaging similar cytopathic effects (CPE) were consistently observed for all 3 viruses. This was also seen when performing the infectious centre assays shown in figure 5. Furthermore, we have now included counts of plaque size in empty vector and ZBP1-expressing HT-29 cells (Figure 4B) (see response to point 3 below), and this confirms that neither of the mutant viruses are impaired for plaque formation in empty vector HT-29 cells. This has been added to the manuscript on page 13, lines 279-287.

2. *As the authors described, ZBP1 is considered to be function in the cytoplasm, although capsid proteins of herpesvirus localize in the nucleus mainly. The authors should analyze the colocalization between ZBP1 or RIPK3 and ORF20 in infected cells.*

We did attempt this experiment, however unfortunately could not produce reliable antibody staining with the reagents against human ZBP1 that are commercially available. Nonetheless, staining for VZV ORF20 in infected cells shows that it is readily detectable in the cytoplasm as well as in the nucleus of infected cells (see data below). Others have also reported that transient transfection of ORF20 in cells results in a largely cytoplasmic distribution of the protein and it is thought that binding to other capsid proteins is required to facilitate nuclear entry (Chaudhuri *et al.*, 2008).

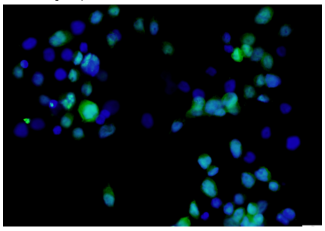

Rebuttal Figure 1. Immunostaining for VZV ORF20 in infected HT-29 cells.
VZV infected HT-29s were immunostained with a mouse anti-ORF20 antibody (Green) and counterstained with DAPI (blue).

Additionally, when co-transfected into 293T cells, GFP-tagged ZBP1frequently can be detected in the same region of the cell as ORF20 (see arrows for examples in figure below).

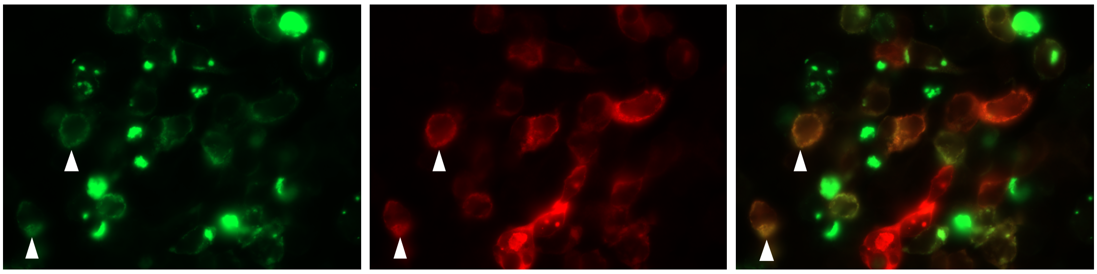

Rebuttal Figure 2. ZBP1-1 and ORF20 localization in 293T cells. 293T cells were co-transfected with expression constructs for ZBP1-GFP and ORF20-V5. At 48 h post-transfection cells were fixed and immunostained using a V5 antibody (red).

Lastly, recently Jiao *et al.*, 2020 (Nature) have shown that ZBP1 and RIPK3 activation can occur in the nucleus of cells, and therefore it is conceivable that VZV ORF20 could interfere with nuclear activation of ZBP1 during capsid assembly as well as in the cytoplasm either when associated with incoming virions or directly after translation. These details have been added to the discussion of the manuscript (page 26-7 lines 615-621)

3. *In this article, the authors argued that the ORF20 RIHM inhibits ZBP1-mediated apoptosis in infected cells but not necroptosis. However, the results that VZV ORF20 interacts with ZBP1 and RIP3 could not explain this difference. Can the ORF20 inhibit necroptosis in transient system? Data in Fig. 4 should be quantified and analyzed statistically.*

Unfortunately, we could not achieve robust transient expression of ORF20 in necroptosis sensitive cells, as explained in the manuscript (page 11, line 230-236).
We have now included quantification and statistical analysis of plaque sizes, included as a new figure 4B and updated the manuscript to reflect this, pages 13-4 (lines 279-287 and 295-300) as well as the associated figure legend page 47 (lines 1109-1112) and materials and methods page 31 (lines 708-710).

Reviewer #2:

1. *The VZV mutants need at least growth curve analyses and comparison on ARPE19 cells*

Please see the detailed response to reviewer 1, question 1.

2. *Also did the authors look at the larger ORF20 deletion mutant for growth and blockade of necroptosis induction? It is possible that just because the AAAA mutant stopped RIP3K interaction in MVMV and HSV, it might not be enough here. This would be more severe than the single four AAAA substitution, which might still interact with RIPK to stop necroptosis through the flanking homology (it can still interact with DAI/ZBP-1….).*

We also considered that a tetra alanine mutation may not be sufficient to stop ORF20 interacting with RIPK3, as this is suggested by the immunoprecipitation and biophysical experiments. Therefore we did also examine TNF necroptosis using a virus which had a full RHIM deletion (20 amino acids) removed. However it showed similar results to the tetra alanine mutant- see below. This extra data has been included in the manuscript on page 12

(lines 256-260) and in supplementary figures S2 B and C, and associated figure legend page 51 (lines 1203-1212).

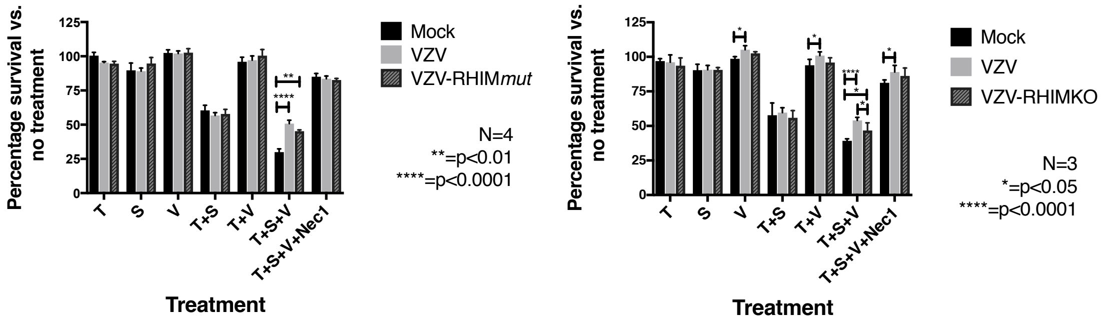

Reviewer #3:

1. *The text that accompanies Figure 1 suggests that VZV sensitized HT-29 cells to TNF-induced apoptosis. While statistics are shown, the text does not accurately reflect the modest sensitization. Here a mechanism would be of interest. Given there is no difference in apoptosis in T+S, does VSV affect cIAP levels? Additionally, the necroptosis inhibition by VSV was modest especially compared to Nec-1 inhibition or inhibition by the ICP6 (Guo et al, 2015) in HT-29 cells.*

We agree that the impact on survival of VZV-infected cells was only modest and have modified the text in line with this suggestion (page 9 lines 195-196). We have examined cIAP1 and 2 levels in VZV infected cells (n=1, see below) and did not detect a difference at the protein level. We have previously reported that VZV impacts TNF signalling by inhibiting the translocation of the p50 and p65 subunits into the nucleus (Sloan *et al.*, 2012), and this may impact cell survival, but remains to be fully investigated.

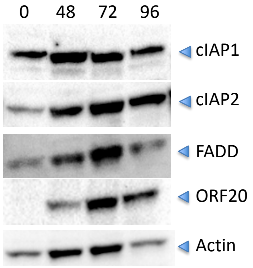

Rebuttal Figure 3. Western blots for components of the TNF signalling pathway following VZV infection. HT-29s were infected with VZV rOKA for the times indicated then cell lysates prepared in DISC lysis buffer and Western blotting performed for cIAP1 and 2, FADD, VZV ORF20 and actin as a loading control.

We also agree that the protection against necroptosis conferred by VZV is modest compared to what has been reported for HSV, however we believe this is due to the complicated nature of VZV infection *in vitro*. By using cell free HSV for infections it is possible to achieve a very high percentage of cells infected. However, with *in vitro* VZV infections being limited to cell-associated, asynchronous infections it is difficult to achieve a similar high percentage of cells infected and therefore protected from necroptosis. This is reflected in the supplementary

figure S2 which shows on average 40% of cells are antigen positive when performing the assays.

2. *In Figure 2, it is unclear how VZV infection conferred resistance to necroptosis in neighboring uninfected cells. Does ORF20 "RHIM" mutation influence VSV replication/infectivity?*

Whilst the bystander cells did show fewer MLKL positive cells compared to mock, there was no statistical difference between the two. It is also possible that some of the bystander cells are actually in the early stages of infection but not yet expressing detectable amounts of viral antigen which may account for the difference. When the ORF20 RHIM was mutated and virus recovered, the mutant virus was always passaged and grew to rates identical to the parent viral strain which was handled in parallel, so we do not believe the RHIM influenced the replication or infectivity of the virus. Also see response to reviewer 1, question 1.

3. *Figure 4 indicates that ZBP1 expressing HT29 cells form fewer infectious centers than parental HT29 cells and that this could be modulated by caspase inhibition but not the necroptosis inhibitor NSA. Western blots or IF for activated caspases should be incorporated to make certain zVAD rescue is on target.*

Whilst we have not performed Western blots for cleaved caspase 3 on ZBP1-expressing HT-29s, we have done so for regular HT-29s and confirmed that z-VAD blocks the cleavage of caspase 3 (see data below).

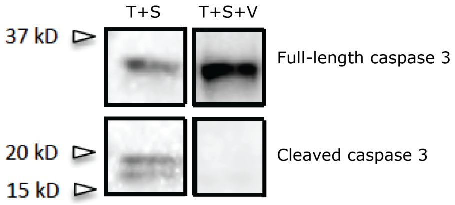

Rebuttal Figure 4. Western blots for caspase 3. HT-29s were treated with T+S to induce apoptosis and T+S+V to induce necroptosis. The addition of z-VAD (V) blocked the cleavage of caspase 3.

4. *Additionally, ZBP1-induced apoptosis occurs via caspase 8 activation via RIPK3-RIPK1 during Influenza infection. Does Nec-1 rescue? Does KO or knockdown of RIPK3 and RIPK1 rescue? FADD dominant negative? ZBP1-expressing HT-29 cells are very sensitive to Influenza (PR8) and HSV1-ICP6mutRHIM-induced necroptosis. Are VSV-infected ZBP1 HT29 cells resistant to ZBP1-induced necroptosis by either Influenza or mutant HSV1?*

These are all interesting questions which we would like to address in the future, however we feel they are outside the scope of this current manuscript and feel that the field would be best served by a detailed, additional study.

5. *The binding assays presented in Figure 6 demonstrate binding of ORF20 to ZBP1 and RIPK3 independent of the ORF20 RHIM core. Inclusion of RIPK1 and TRIF would be informative. Mutation of the RHIM domain in RIPK3 and ZBP1 should be shown. The ORF20 RHIM-KO similar to Figure 4 or other mutation that eliminates binding should be identified.*

We sought to examine interactions in cells between ORF20 and RIPK1 and were unable to detect co-immunoprecipitation despite multiple attempts. We have now included data from single molecular confocal spectroscopy experiments in Supplementary Figure S4A (Page 18 line 402-409) that also indicate that ORF20 does not interact with RIPK1 (Figure legend page 50 lines 1220-1223).

We have previously demonstrated that AAAA mutation of the RIPK3 RHIM does not completely abrogate oligomerisation of RIPK3 (Pham et al. 2019 EMBO Reports) but does reduce the stability of the oligomers. Identification of the site of interaction between ORF20 and RIPK3 is beyond the scope of this current work.

We have now included data in Supplementary Figure 4B in-text discussion page 19 lines 426-435 and figure legend page 50 (lines 1223-1228) that reports on the effect of mutation of the two RHIMs in ZBP1 on its interaction with ORF20. We observe that the interaction with ORF20 occurs through the RHIM closest to the N-terminus of ZBP1.

Minor issues raised:

Reviewer #1:
1. *In Fig. 2B, please show the results of mock with and without TSV.*

All results shown in Fig 2B are for TSV treated cells. Cells without TSV showed no pMLKL as shown in Fig 2A. We believe that including no TSV for the mock in 2B may confuse the reader.

2. *In the legend of Fig. 3, please indicate time after infection.*

This has been added to Figure 3 page 45 line 1097.

3. *In Fig. 4 and 5, please indicate a strain name instead of Parent in the label.*

This has been corrected in both figures and associated figure legends (page 45 line 1105 and page 45 line 1117, respectively).

4. *Please indicate the protein detected for infection in Fig. S1C.*

This has been added.

Reviewer #2:
1. *The HSV capsid protein is not included in figure 1 alignment. Is there any sign of a RHIM like motif in it? Secondly, is it detectable in the Beta and Gamma herpesvirus equivalents.*

There is no RHIM-like motif in the capsid triplex 1 of HSV-1 or -2 (VP19C), nor could we find any in the human beta or gamma herpesvirus equivalents. This has been added to the manuscript on page 22, lines 512-514.

2. *Fig1c. Cell images appear to be different sizes and magnifications and need a size bar-*

Scale bar information has been added to the figure legend page 45 lines 1111-1112.

3. *The VZV mutants need at least growth curve analyses and comparison on ARPE19 cells*

Please see our response to reviewer 1, question 1 above.

4. *Line 190 how long past infection were the studies done? And the level of necroptosis seems rather weak, and the effect of inhibition is modest. Also did the authors look at the larger ORF20 deletion mutant for growth and blockade of necroptosis induction? It is possible that just because the AAAA mutant stopped RIP3K interaction in MVMV and HSV, it might not be enough here. This would be more severe than the single four AAAA substitution, which might still interact with RIPK to stop necroptosis through the flanking homology (it can still interact with DAI/ZBP-1….).*

The necroptosis experiments were performed at 72 h post-infection and this information has now been added to the text (page 9, line 190 and figure legend (page 44 line 1076 and page 45 line 1079). Please see detailed response above, Reviewer 2, point 2 and Reviewer 3, point 1.

5. *Figure 2A could be a lot more convincing. The cells showing MKLK and much less dense and the red signal is barely visible. Indeed, the correlation with VZV and uninfected cells could be improved by showing flow plots and the red and green signals in black and white, separate channels rather than overlap. Why is the MKLK only being activated in 20% of cells? this seems rather low, and others using HRT1080s have gotten much higher levels.*

This is the response we saw at 7-8 hr post-treatment, it is possible that with longer treatment the percentage of pMLKL would increase however we wanted to ensure we did not lose too many cells due to detachment from the coverslip.

6. *S1C showing P-MKLK should go into the main figures. Also why is MKLK missing from two lanes in total assessment levels?*

Total MLKL is very low in two lanes due to low levels of protein added which is reflected in the actin stain. This is why we preferred to keep this as a supplementary image.

7. *Lin 265 What are the endogenous levels of ZBP1 in the cells transfected? how much higher levels are they needing to see the effect?*

HT-29s do not express endogenous ZBP1 and must be transduced to achieve expression (Guo *et al.*, 2018).

8. *Given that the mutant ORF20 proteins still form a complex with ZBP-1 even though it is rather atypical, why does it still not inhibit apoptosis? A bit more discussion is needed*

It was interesting to note that, unlike other reported viral RHIM proteins that inhibit programmed cell death, a RHIM tetra-alanine mutant form of ORF20 was still able to interact and form heteromeric structures with ZBP1 and RIPK3. However, these had a morphology unlike the fibrillar complexes formed with WT proteins. Additionally, the complexes formed between $ORF20_{1\text{-}114mut}$ and $ZBP1_{170\text{-}355}$ were much less stable than those formed by wild-type ORF20 with ZBP1. The high resolution structure of the host RIPK1-RIPK3 RHIM amyloid necrosome fibril core demonstrates that specific and selective residue-residue interactions define the stoichiometry and morphology of host necrosome structures (Mompean et al. 2018 Cell). We have recently shown that an AAAA RHIM mutant of RIPK3 can also form an amyloid-type structure *in vitro* (Pham et al. 2019 EMBO Reports). Hence we conclude that in the

ORF20-RHIMmut, as in RIPK3-RHIMmut, residues outside the core tetrad can form inter-protein contacts to generate complexes with elements of cross-β structure. However, these mutant RHIM structures lack the ordered, specific and highly stable fibrillar array of the WT amyloid fold that is capable of either signalling (for host proteins) or ablating signalling (for viral proteins). That is, the wild type complex formed by ORF20 and ZBP1 has a particular stable

and ordered fibrillar architecture that sequesters ZBP1 and prevents the latter from signalling, while the complex formed between the ORF20 mutant and ZBP1 does not stably inhibit ZBP1 function.

We have expanded the discussion of this point in the manuscript (page 25-26 lines 582-598).

Reviewer #3:

1. *Acknowledging, the challenge of transducing HT29 cells with a ORF20 expression construct, does ORF20 expression in other necroptosis-sensitive cells such as murine L929 or SVEC4-10 or ZBP1/RIPK3 transduced 293T or HeLa suppress necroptosis/necroptosis?*

We have attempted these experiments using Hela/293T expression systems but never achieved a high enough simultaneous expression of RIPK3 and ORF20 to achieve reproducible results. Given the strict species-specificity of VZV infection we felt that examining non-human cells was not within the scope of this manuscript, however this point remains of interest for the future.
